# Safe at home: prevention of pediatric unintentional injuries

**DOI:** 10.1186/s40621-023-00442-9

**Published:** 2023-07-03

**Authors:** Coleman Burch, Alicia Webb, Eric Jorge, Bill King, Michele Nichols, Kathy Monroe

**Affiliations:** 1 University of Alabama Heersink School of Medicine, Birmingham, USA; 2 Division of Pediatric Emergency Medicine, Department of Pediatrics, University of Alabama Heersink School of Medicine, Birmingham, USA

**Keywords:** Children, Firearms, Ingestions, Injury, Medications, Prevention, Safety, Storage

## Abstract

**Background:**

Unintentional injuries are the leading cause of death in children in the United States. Studies have shown that parent adherence to safety guidelines is improved when education is provided in conjunction with safety equipment.

**Methods:**

This study surveyed parents about specific injury prevention behaviors regarding medication and firearm storage and provided education and safety equipment for safe practice of these behaviors. The project took place in a pediatric emergency department (PED) and partnered with the hospital foundation and the school of medicine. Inclusion criteria were families visiting a freestanding PED in a tertiary care center. Participants completed a survey conducted by a medical student approximately 5 min in length. The student then provided each family with a medication lock box (if children ≤ 5 years old lived in the home), firearm cable lock, and education for safe storage of medications and firearms in the home.

**Results:**

The medical student researcher spent a total of 20 h in the PED from June to August 2021. 106 families were approached to participate in the study, of which 99 agreed to participate (93.4%). A total of 199 children were reached with ages ranging from less than 1 year old to 18 years old. A total of 73 medication lockboxes and 95 firearm locks were distributed. The majority (79.8%) of survey participants were the mother of the patient and 97.0% of participants lived with the patient > 50% of the time. For medication storage, 12.1% of families store medications locked and 71.7% reported never receiving medication storage education from a healthcare professional. Regarding firearms, 65.2% of participants who reported having at least 1 firearm in the home stored firearms locked and unloaded with various methods of storage. 77.8% of firearm owners reported storing ammunition in a separate location from the firearm. Of all participants surveyed, 82.8% reported never receiving firearm storage education from a healthcare professional.

**Conclusions:**

The pediatric ED is an excellent setting for injury prevention and education. Many families are not storing medications and firearms safely, demonstrating a clear opportunity to increase knowledge in families with young children.

## Background

Unintentional injury remains the leading cause of death among children ages 1 to 18 in the United States [CDC, Fatal injury data (WISQARS)]. In 2020, there were over four million unintentional injuries and more than 8000 unintentional deaths according to the most recent report [CDC, Fatal injury data (WISQARS)]. Among the leading causes of death in children were medication poisoning and firearms, ranking at numbers two and nine, respectively [CDC, Fatal injury data (WISQARS)]. It is estimated that unintentional deaths caused by firearms and medication poisoning cost the U.S. healthcare system over $20 billion annually [CDC, Cost of injury data (WISQARS)]. Given that these injuries and deaths are both costly and preventable, it is critical to reduce the cost, morbidity, and mortality in the vulnerable pediatric population.

Unintentional firearm injuries and fatalities in children can be prevented when caretakers are educated about firearm safety and storage. Effective practices include storing firearms locked, unloaded, and storing ammunition in a locked place away from firearms (Grossman et al. 2005; U.S. Department of Veterans Affairs and American Foundation for Suicide Prevention 2022). Simple firearm locks, such as trigger locks and cable locks, are relatively inexpensive and easy to use and can be bought at most local retailers. However, many households do not practice the safest form of gun storage. One study suggested that 1 in 3 households contain at least one firearm and of these households only 3 in every 10 practice the safest form of gun storage (Azrael et al. 2018).

It is believed that rates of unintentional medication poisoning in children can be reduced by educating caregivers and providing safe and effective medication storage equipment. Safe medication storage includes storing medication in a locked box or container out of the reach of children (CDC, Patient Safety 2022).

Given that the pediatric emergency department (PED) is where many of these injuries undoubtedly present, it is only fitting for education of injury prevention to take place in the PED as well. PED staff are well equipped and knowledgeable in injury prevention; however, taking time to educate families in a busy PED environment may be seen as a burden to staff and an interruption to workflow. Additionally, many patients and caregivers often have downtime in the PED while waiting to receive care, presenting an additional opportunity for injury prevention education by a designated staff member.

While previous studies have shown a need for educating caregivers, little work has been done to demonstrate effective injury prevention programs in the PED by a staff member dedicated to injury prevention efforts. Previous work has shown that patient adherence is improved when education is paired with safety equipment that families can use (Kendrick et al. 2009, 2012). A prior study at our PED in 2019 partnered with the School of Public Health (SPH) and surveyed caregiver practices regarding medication storage, firearm storage, and other safety practices while also providing safety equipment to participants (Webb et al. 2022). This study demonstrated that caregivers often report unsafe storage of medications and firearms and showed effective outreach by partnering with local schools and resources.

### Objectives

1. To measure the self-reported safe storage practices of medications and firearms in the home.
2. To compare changes in the safe storage practices between the 2019 and 2020 surveys.
3. To evaluate the responsiveness of obtaining survey data from patient families in the pediatric emergency department.
4. To provide descriptive data of the respondents from the 2020 survey.

## Results

The PI spent a total of 20 h in the PED from June to August 2021. 106 families were approached to participate in the study, of which 99 agreed to participate (93.4%). A total of 199 children were reached with ages ranging from less than 1 year old to 18 years old. The average age of children per household was 6.6 years. A total of 73 medication lockboxes and 95 firearm locks were distributed. The majority (79.8%) of survey participants were the mother of the patient and 97.0% of participants lived with the patient more than 50% of the time (Table 1)*.*

**Table 1:** Survey responses

*1. What is your relationship to the patient?*	
*Mother*	79 (79.8%)
Father	9 (9.1%)
Grandmother	6 (6.1%)
Grandfather	2 (2.0%)
Other	3 (3.0%)
*2. Do you live with the patient at least 50% of the time?*	
Yes	96 (97.0%)
No, less than 50% of the time	1 (1.0%)
No, I don’t live or stay with the patient	2 (2.0%)
*3. How many children (less than 18 years of age) do you routinely provide care for or live with?*	
Total	199 (range 0–5)
*4. What are the ages of the children you routinely provide care for?*	
Mean	6.6 years (range < 1 to 18)
*5. How do you currently store your medications?*	
In a locked box, container, cabinet, or drawer	12 (12.1%)
In an unlocked box, container, cabinet, or drawer	75 (75.8%)
On top of a dresser/counter/table/nightstand	21 (21.1%)
In the refrigerator	9 (9.1%)
In a purse/bag	4 (4.0%)
Other	0 (0%)
*6. Has a health care professional ever discussed safe storage of medications with you?*	
Yes	28 (28.3%)
No	71 (71.2%)
*7. How do you store the firearm(s) in your home?*	
Locked and unloaded	30 (30.3%)
Locked and loaded	11 (11.1%)
Unlocked and unloaded	7 (7.1%)
Unlocked and loaded	3 (3.0%)
Not applicable	53 (53.5%)
*8. How many firearms are in the home?*	
Mean	1.6 (range 0–20)
*9. If applicable, how do you lock your firearms?*	
Trigger lock	2 (4.8%)
Cable lock	2 (4.8%)
Gun lockbox	18 (42.9%)
Biometric (fingerprint) lock	3 (7.1%)
Other	21 (50%)
*10. If applicable, do you store firearms and ammunition separately?*	
Yes	35 (77.8%)
No	10 (22.2%)
*11. Has a health care professional ever discussed safe storage of firearms with you?*	
Yes	17 (17.2%)
No	82 (82.3%)

### Medication storage

Of those surveyed, 12.1% of families reported storing medications in a locked location and 71.7% reported never receiving medication storage education from a healthcare professional.

### Firearm storage

Of those who admitted to firearm ownership, 59% of participants reported storing their firearms locked and unloaded. Additionally, 77.8% of firearm owners reported storing ammunition in a separate location from the firearm. The majority of firearm owners reported using either a gun lockbox (42.9%) or other forms of storage (50%). Of all participants surveyed, 82.8% reported never receiving firearm storage education from a healthcare professional.

### Survey comparisons

Two important comparisons were made between this survey and the previous survey completed in our PED: (1) Medication storage “in a locked box, container, cabinet, or drawer” response improved from 9% of respondents to 12% of respondents, but failed statistical significance (*z* =  − 0.9, *p* = 0.4); (2) Of those who admitted to firearm ownership, storing firearms “locked and unloaded” improved from 45 to 59% but did not reach statistical significance (*z* =  − 1.7, *p *= 0.08).

## Discussion

Our study demonstrated rates of safe medication (Webb et al. 2020; Mohr et al. 2021) and firearm (Aitken et al. 2020; Jennissen et al. 2021; King et al. 2020) storage similar to previous studies, including the previous one conducted at this institution with the SPH. Additionally, we reported the recall rates of previous education by a healthcare professional to be alarmingly low for both medication and firearm storage. Given reported low education rates, there would seem to be a disconnect between providing safety education and caregivers retaining this information, but it is unclear where the disconnect lies. One study analyzed audiotapes of pediatric visits in an urban clinic and found that 36% of injury prevention counseling discussed ingestions, aspirations, and suffocations, the most of any topic, while zero discussed firearm safety or prevention strategies (Gielen et al. 1997). Many patient families, separate from our survey, stated they had received firearm safety education from local law enforcement, or another organization experienced with firearms. PEDs could potentially benefit from partnership with these organizations. More studies are needed to formally assess families’ attitudes and experiences regarding firearm education from these organizations.

Compared to the 2019 study, there was no statistically significant increase in the rate of safe firearm or medication storage (Fig. 3). However, our study demonstrated a more efficient and effective patient education process, reaching more families and children in less time (Table 2). While the previous study inquired about other injury prevention topics in addition to medication and firearm storage, we solely focused on the latter two topics given their high occurrence and ability to provide equipment to reduce injury. By reducing the survey length and eliminating follow up, we were able to focus on surveying families’ safety practices and provide brief but informative education regarding injury prevention. Both studies demonstrate that PEDs could benefit from a designated injury prevention educator embedded in the PED. While PED physicians are equipped for injury prevention counseling, many are too busy and overwhelmed (Gittelman and Durbin 2005). In addition, patients in PEDs often have downtime waiting for providers, lab results, or imaging during their stay. This provides an opportunity for dedicated injury prevention counselors to educate families as was done in this study. Both studies also demonstrated low self-reported rates of safe firearm and medication storage as well as promising outreach opportunities by partnering with other local resources.

**Table 2:** Comparison of 2019 and current studies

	Sample size	Children reached	% safe medication storage	% safe firearm storage (for those with firearms)	Hours worked	Hours worked per child reached
2019 study	357	843	9	45	180	0.2
Current study	99	199	12	59	20	0.1

In addition, since the PED is where most firearm and ingestion-related injuries present, it is only fitting that education to prevent these injuries should occur in the PED as well. These “teachable moments” should be normalized in the PED setting as previous studies have shown patient education in the PED to be an opportunity worth seizing (Melzer-Lange et al. 2013; Pétré et al. 2020). As discussed earlier, dedicated injury prevention counselors could be valuable in providing education and connecting families to community resources in the midst of a busy PED setting. Future directions include preventing these injuries in adolescent and mental health populations, as well as the rates of intentional injuries of firearms and medication ingestions.

Limitations of this study include reliance on self-reporting of behaviors in addition to the absence of a control group to compare interventions. While behaviors were self-reported, we expect firearm ownership to be underreported and the safe storage of firearms to be overreported given the controversial nature of guns and the high prevalence of gun ownership in our state. In addition, more people accepted a firearm lock when offered (*n *= 95) than those who admitted to gun ownership (*n *= 51). We also acknowledge that verbally administering the survey could dissuade participants to disclose private information. Finally, there was no follow-up component to this study as in the 2019 study, however, our objective was not to survey the satisfaction with the provided safety equipment but instead to survey family storage practices and experience with prior safety education and counseling.

## Conclusion

We found that the rate of safe medication and firearm storage was similar to previous studies in our institution. Many families are not storing medications and firearms safely nor do they recall having received safety education from a healthcare professional. In addition, our study streamlined the patient education process about these topics compared to previous studies and further demonstrates the benefit of a designated worker embedded into the PED as well as normalizing safety discussions with families in the PED itself.

## Methods

**Fig. 1:**
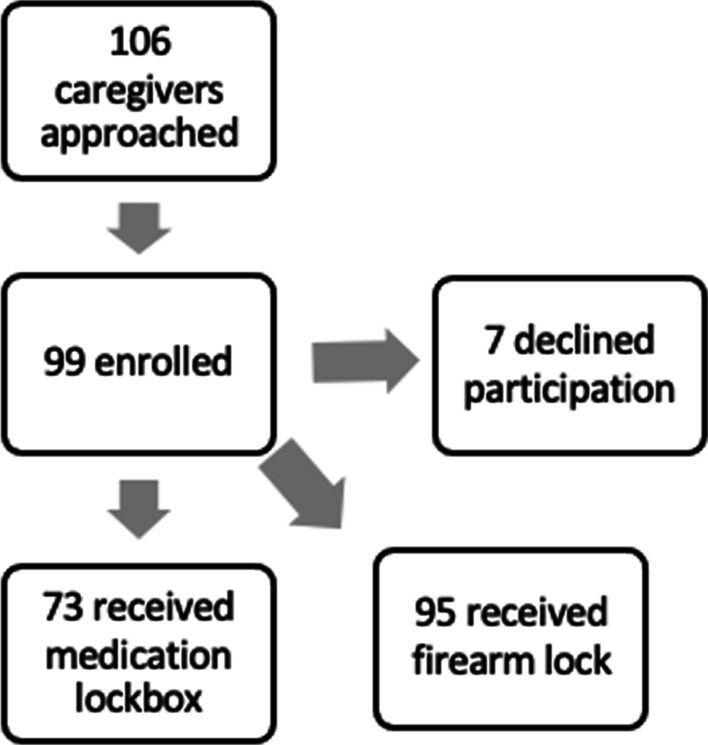
Design of study

A survey about medication and firearm storage was administered to a convenience sample of caregivers presenting to our freestanding pediatric tertiary care hospital in an urban city center. The survey questions were verbally read to the participants in a private room and responses recorded by the PI. Following the survey, the principal investigator (PI) provided the caregiver with verbal and written education for safe storage of medications and firearms in the home along with a medication lock box (for those with children five years of age and younger in the home) and a firearm cable lock for all participants (Fig. 1). In this pilot study with limited funding, the age cutoff was selected to focus more on unintentional ingestions in younger children which is more prevalent in our institution’s population. The study partnered with the Children’s hospital foundation (funding for equipment) and the School of Medicine (PI’s time).

Inclusion criteria were English-speaking caregivers of patients < 19 years old presenting to the PED with an Emergency Severity Index (ESI) score of 3, 4, or 5. Caretakers of a patient who presented with a mental health concern were excluded as were caretakers of children meeting critically ill ESI level 1 or 2 criteria. At our institution, many psychiatric chief complaints are automatically classified as ESI ≤ 2 and thus were excluded. We defined safe firearm storage as storing firearms locked and unloaded; we defined safe medication storage as storing medication in a locked box, container, or other device.

### Survey

**Fig. 2:**
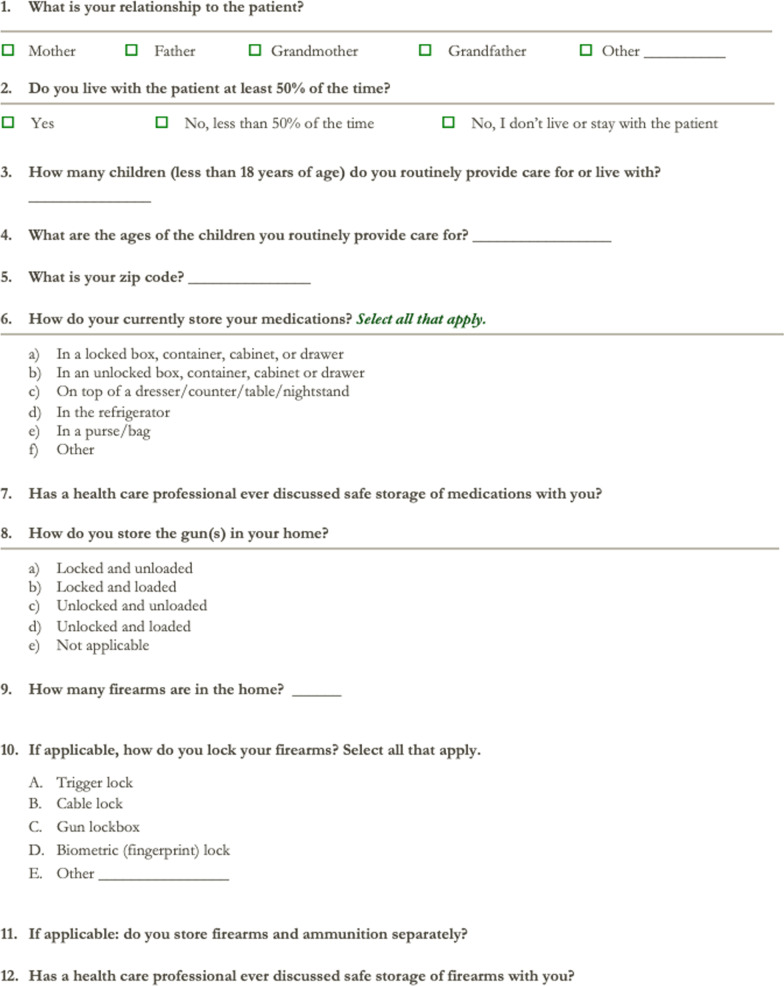
Survey administered to participants

Participants verbally completed an online survey conducted by the PI that was approximately five minutes in length. The survey assessed the participant’s relationship to the patient, number of children living in the home, zip code of residence, current firearm and medication storage practices at home, and previous firearm and medication storage education received (Fig. 2)*.* Consent to participate was confirmed, and participation was confidential and voluntary. Answers were recorded in Survey Monkey^®^.

### Education and equipment

**Fig. 3:**
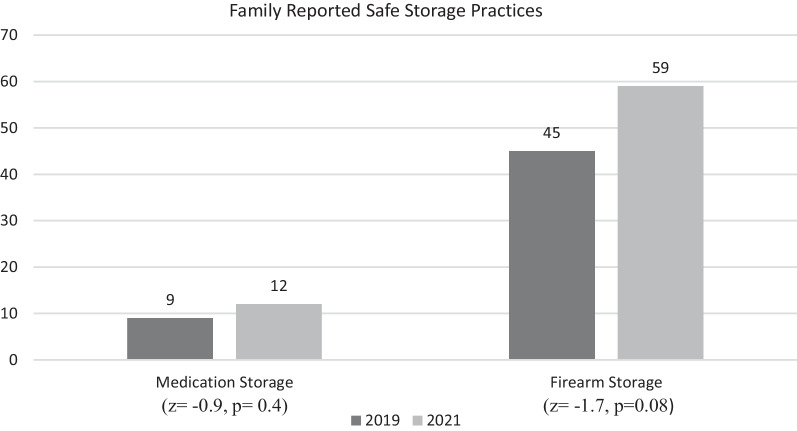
Medication and firearm safe storage practices

The PI provided each family who completed the survey with a medication lock box if children 5 years old or younger lived in the home and a firearm cable lock for all participants. Verbal education and written handouts on safe storage of medications and firearms in the home were also provided (Fig. 3).

### Statistical methods

Comparison of the current study sample results versus the 2019 study sample was accomplished using the Z test of population proportions (comparing proportions of respondents practicing safe storage practices of firearms and medications). The two independent samples were comprised of our hospital PED patient families, who were recruited during two different time periods.

## Data Availability

The datasets used and/or analyzed during the current study are available from the corresponding author on reasonable request.

## Abbreviations

ESI: Emergency Severity Index
PED: Pediatric emergency department
PI: Principal investigator
SPH: School of Public Health
